# Case Report: Paratesticular Rhabdomyosarcoma

**DOI:** 10.3389/fonc.2021.629878

**Published:** 2021-03-17

**Authors:** Yiyi Zhu, Ziwei Zhu, Yunyuan Xiao, Zaisheng Zhu

**Affiliations:** ^1^Department of Endocrinology, Peking Union Medical College Hospital, Peking Union Medical College, Chinese Academy of Medical Sciences, Beijing, China; ^2^Department of Urology, Jinhua Hospital Affiliated to Zhejiang University School of Medicine, Jinhua, China

**Keywords:** paratesticular rhabdomyosarcoma, nerve-sparing retroperitoneal lymph node dissection, chemotherapy and radiotherapy, sperm cryopreservation, endocrine follow-up

## Abstract

Paratesticular rhabdomyosarcoma (RMS) accounts for only 7% of all the RMS cases. Due to the limited available data, there is no consensus on the diagnosis and management of the paratesticular tumors. Here, we interrogated two paratesticular RMS cases in 25 and 27-year-old men presenting with painless and rapidly growing mass in the scrotum. Whereas the data showed no upregulation of tumor markers such as *β*-human chorionic gonadotropin (*β*-HCG), alpha-fetoprotein (AFP), and lactate dehydrogenase (LDH), scrotal ultrasonography and magnetic resonance imaging indicated the existence of paratesticular and inguinal lesions respectively. There was local recurrence in one patient who underwent radical orchiectomy for the sarcoma one year ago. In addition, the CT scans showed no occurrence of distant metastasis. The two patients underwent radical inguinal orchiectomy or resection of the recurrent tumors with nerve-sparing retroperitoneal lymph node dissection. Histologic examination revealed embryonal RMS (eRMS) without lymph node metastasis. We highlight the importance of multi-disciplinary participation for paratesticular RMS detection and preoperative ultrasound-guided needle biopsy (UNB) for rapid confirmatory diagnosis. Complete surgical resection coupled with chemotherapy and radiotherapy is the main treatment option for the paratesticular RMS. In addition, sperm cryopreservation treatment and endocrine follow-up could increase the overall survival and quality of life of the patients.

## Case Presentation

### Case 1

**Figure 1:**
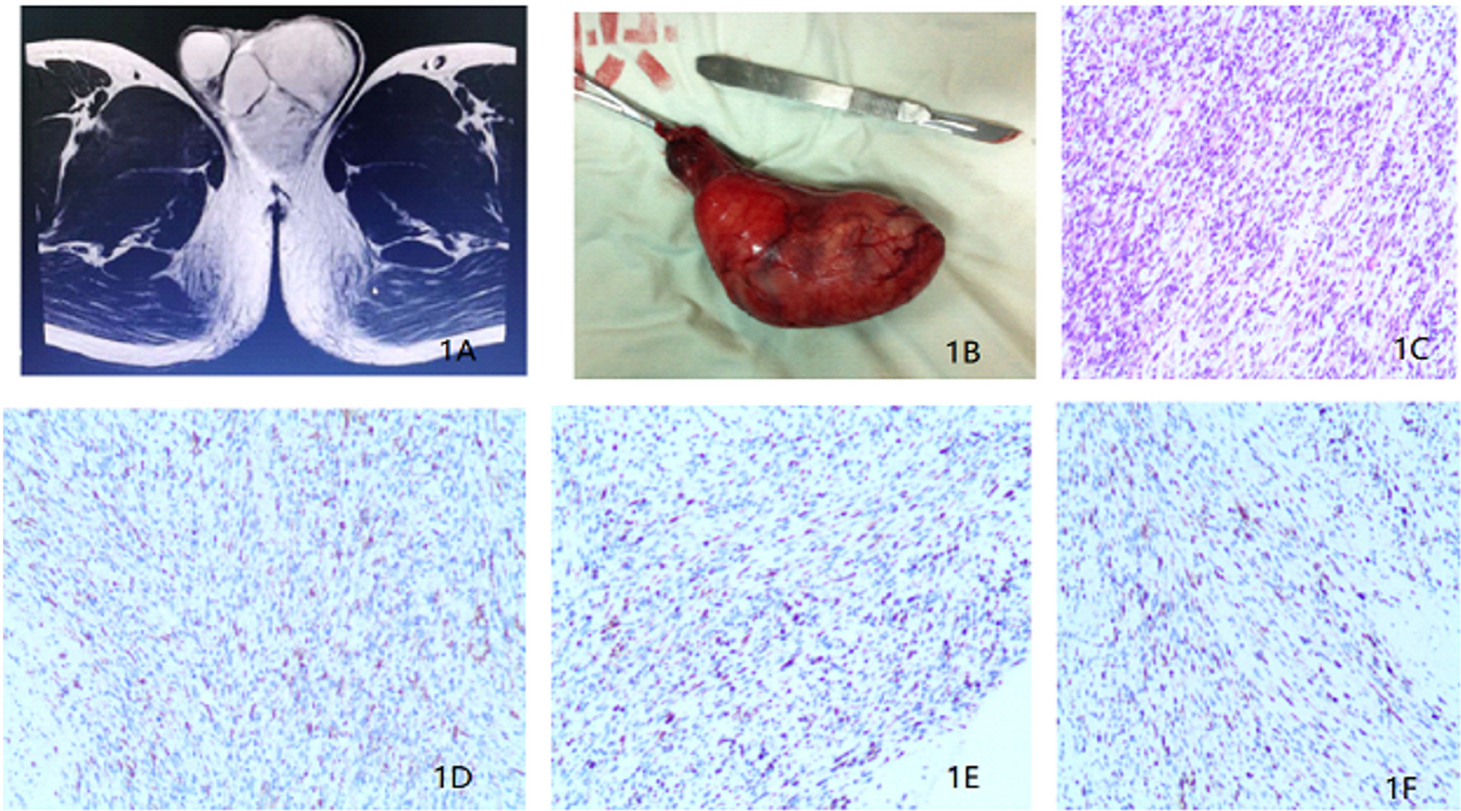
**(A)** Magnetic resonance imaging revealing a soft tissue mass in the left scrotum. **(B)** Surgical piece of the left orchiectomy presenting a 9.5 × 5.0 × 4.5 cm^3^ paratesticular tumor. **(C)** Histopathological section (hematoxylin and eosin staining). **(D)** Immunohistochemistry results showing the expression of Desmin. **(E)** Immunohistochemistry results showing the expression of MyoD1. **(F)** Immunohistochemistry results showing the expression of Myogenin.

In Jan 2018, a 25-year-old unmarried man with no past medical or family cancer history presented to the hospital with a progressively growing but painless swelling in his left hemi-scrotum. The swelling had lasted for one month. Physical examination confirmed the swelling measuring about 9.0 × 5.0 × 6.0 cm, and the light transmittance test was negative. It was a painless and rubbery mass with poorly defined edges. Interestingly, all the known and relevant tumor markers such as cancer-associated antigen 19-9 (CA19-9), carcinoembryonic antigen (CEA), alpha-fetoprotein (AFP), *β*-human chorionic gonadotropin (*β*-HCG) and lactate dehydrogenase (LDH) were within normal ranges. Scrotal ultrasonography demonstrated an irregular and isoechoic mass within the left hemi-scrotum, while the testicular size was normal. In addition, Color Doppler ultrasound showed significant vascularity within the mass and a resistance index (RI) of 0.71, indicating a high probability of a tumor. On the other hand, magnetic resonance imaging (MRI) showed that the soft tissue mass in the left scrotum was closely associated with the epididymis and had gradual enhancement after contrast suggesting that the lesion might be arising from mesenchymal tissues (**Figure 1A**). A CT scan for the chest, abdomen, and pelvis did not indicate evidence of metastasis. For a definitive diagnosis without post-operative bleeding and tumor implantation along the needle track, preoperative ultrasound-guided needle biopsy (UNB) using a coaxial needle was adopted. The pathologic data from the UNB demonstrated presence of a myxoid stroma with a rich vascular weave in paratesticular tissue samples. Spindle and irregular cells were arranged in parallel or in a mesh-like pattern with abundant red-staining cytosol. There was a significant presence of mitotic bodies. Besides, immunohistochemical (IHC) experiments detected Ki-67 and Desmin, but not S-100, CEA, CD34, keratin and Caldesmon-H. A multi-disciplinary team (MDT) of experts from urinary surgery, medical oncology, endocrinology, reproductive health, radiotherapy, medical imaging and pathology departments concurred on the use of radical inguinal orchiectomy, coupled with chemotherapy and radiotherapy to the retroperitoneal lymph node region. During the operation, a pear-like lesion could be seen on the cauda epididymis with a complete outer membrane (scrotal sheath) and a diameter of 9.5 × 5.0 × 4.5 cm (**Figure 1B**). After the operation, the patient recovered without any complication. The postoperative pathological examination confirmed eRMS (**Figure 1C**) and a negative surgical margin. In addition, IHC analysis revealed the expression of Desmin, MyoD1 and Myogenin in the excised tissue samples (**Figures 1D–F**).

Based on the staging evaluation by Intergroup Rhabdomyosarcoma Study Group (1), the case was classified as T1 clinical stage. Since there was no standard chemotherapy regimen for adult paratesticular RMS, the patient was administered with a pediatric VAC regimen comprising of vincristine, actinomycin D and cyclophosphamide (2, 3). Before chemoradiotherapy, he was referred to the Women’s hospital, Zhejiang University School of Medicine for sperm cryopreservation from 8 ml of semen. He then received adjuvant radiotherapy of 30 Gy (retroperitoneal area) and 20 Gy (tumor bed) and six cycles of adjuvant chemotherapy. Whereas the patient reported side effects such as pancytopenia, vomiting, loss of appetite, and hair-loss during chemoradiotherapy, he completed the treatment plan. The patient was finally discharged from the hospital in a tumor-free state. However, he was given traditional Chinese medicine named Guilingji combined with HCG treatment scheme (1 500 IU intramuscular injection once every two weeks) as maintenance therapy. The patient underwent follow-up schedules with scrotal ultrasound in the 6^th^ month, CT scan in the 12^th^ month, and positron emission tomography-computed tomography (PET/CT) in the 24^th^ month. Over the 24-month follow-up period, he remained free from local recurrence and distant metastases. The last physical examination showed restoration of his erectile functions as well as serum endocrine hormones such as follicle-stimulating hormone (FSH), luteinizing hormone (LH), estrogen (E), and testosterone (T). However, two semen examinations indicated oligospermia.

### Case 2

**Figure 2:**
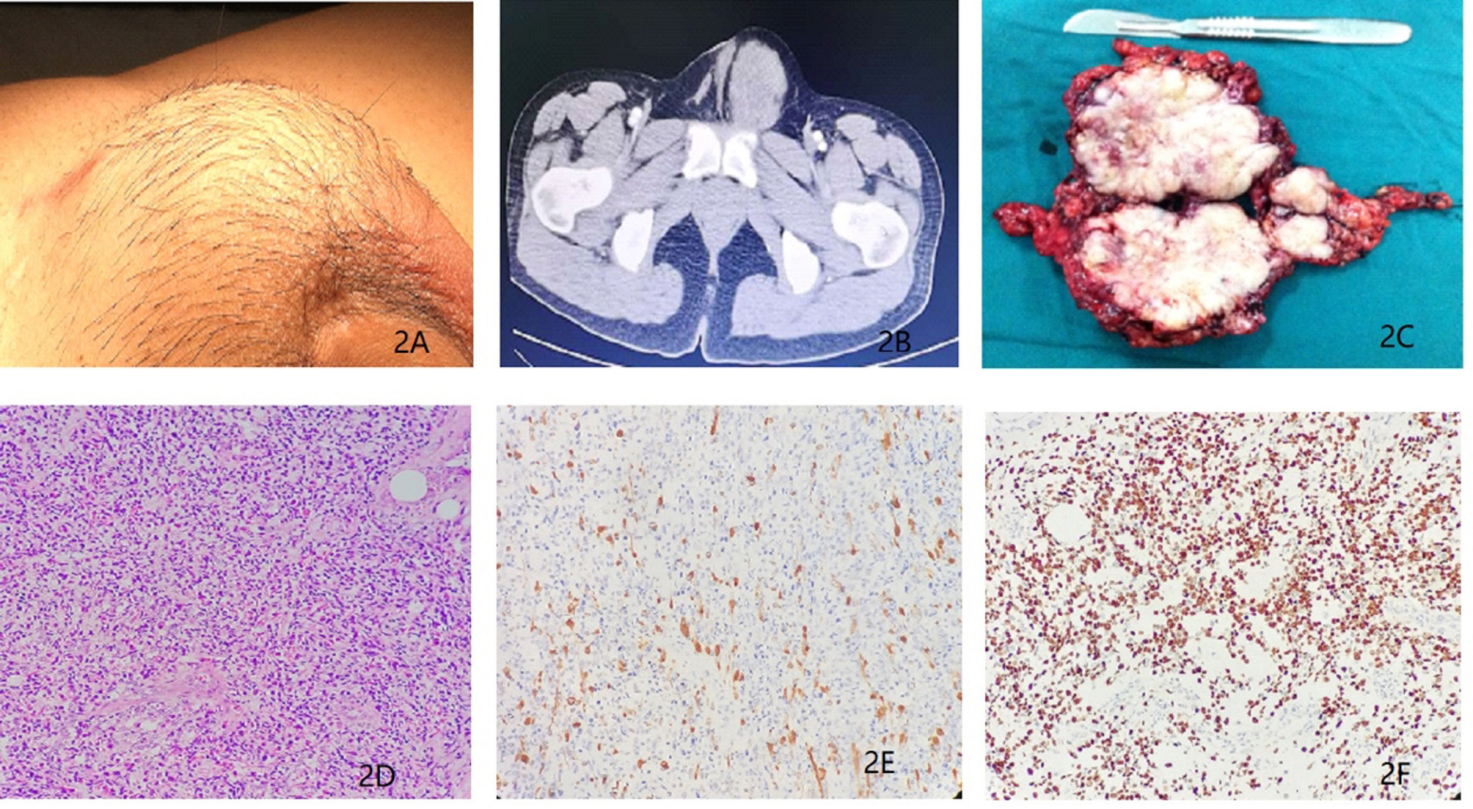
**(A)** Recurrent tumors in the left inguinal region for a duration of 1 month. **(B)** Magnetic resonance imaging revealing presence of a soft tissue mass above the left groin and pelvis. **(C)** Surgical biopsy of the recurrent tumors from the left inguinal and pelvic region **(D)** Histopathological section (hematoxylin and eosin staining). **(E)** Immunohistochemistry results showing the expression of Desmin. **(F)** Immunohistochemistry results showing the expression of MyoD1.

In Dec 2015, a 27-year-old man came to our department with a history of orchiectomy to the left testicular mass. The surgery was conducted a year ago. The patient reported to a urologist and complained of presence of a mass in the left inguinal region that persisted for 1 month. Previously, he was diagnosed with a paratesticular tumor and underwent radical orchiectomy in a different hospital. The postoperative pathological diagnosis showed eRMS (5 cm in diameter) and no adjuvant therapy was administered post-surgery. On physical examination, a surgical scar in the left inguinal region and a 4.0 × 5.0 cm mass localized above the inner ring of the inguinal canal were palpable. This mass was hard and immobile, growing into the pelvic cavity (**Figure 2A**), but superficial lymph nodes were normal in size. His routine laboratory data such as complete blood cell count, renal and liver function tests were normal. The serum tumor markers such as CEA, AFP, *β*-HCG and LDH showed no significant abnormalities. Besides, a scrotum echography showed a heterogeneous mass above the left groin and pelvis. In addition, chest, abdominal and pelvis CT and MRI scans showed an irregular lesion above the left groin and pelvis (**Figure 2B**), indicating tumor recurrence and metastasis. There was, however, no evidence of metastasis to either the lymph nodes or other organs. After MDT discussion and the patient’s permission, tumor resection was performed in combination with adjuvant chemotherapy. Before chemotherapy, the patient underwent sperm cryopreservation at the reproductive center of Renji Hospital, Shanghai Jiaotong University School of Medicine. Similarly, pediatric VAC regimen was applied with vincristine 1.5 mg/m^2^, actinomycin D 1.5 mg/m^2^, and cyclophosphamide 500 mg/m^2^. Only three cycles of chemotherapy were administered due to the side effects, and then the patient received nerve-sparing RPLND. Postoperative pathologic examinations showed that the gross specimen had no obvious capsule and had a pale profile with a fish-flesh appearance (**Figure 2C**). In addition, the examination revealed that the recurrent tumor was eRMS and resection margins were negative (**Figure 2D**). No metastases were found in the lymph nodes. Microscopically, the mass cells were poorly differentiated while the rhabdomyoblasts had immense eosinophilic cytoplasm and necrotic tissues. The immunostaining results showed the expression of Desmin and MyoD1 in the tumor cells (**Figures 2E, F**). Thereafter, adjuvant radiotherapy (30 Gy) was delivered to the tumor bed. To restore reproductive functions, the patient was prescribed with traditional Chinese medicine named Guilingji, HCG treatment scheme (1,500 IU intramuscular injection once every two weeks) and sildenafil, 3 months following the surgery. The patient’s erectile function gradually recovered and semen activity increased after nine months of surgery. The patient underwent regular follow-up (twice every year) by clinic visits and was monitored by scrotal ultrasound, abdominal CT scan and serum endocrine hormones. The last follow-up was 39 months after the operation, and the PET-CT did not reveal any local recurrence or distant metastases. His erectile function and semen analysis returned to normal range. At present, his family moved to another city (Guangzhou, China) with a newborn baby girl.

## Discussion

### Rapid Confirmatory Diagnosis

Compared with other intra-scrotal tumors, the clinical manifestations of paratesticular RMS are non-specific. When there is difficulty in physical examination of tumor localization, ultrasonography (US) can be a useful alternative in the diagnosis (4). The scrotal US results indicated that the lesions were heterogeneous echogenic mass and were extended to scrotum and inguinal region (5). In addition, the Color Doppler ultrasound image showed increased blood flow inside the mass which help discriminate testicular from paratesticular localization (6). Previous studies reported that US of the scrotum and its contents can reliably distinguish testis and scrotal masses, with a sensitivity of >95% (7). Whereas both CT scan and MRI can accurately evaluate the location, size and metastasis of the mass, they cannot be used as confirmatory diagnostic tools. Differential diagnosis of paratesticular RMS includes lesions such as leiomyosarcoma, liposarcoma and fibrosarcoma. These tumors lack imaging features, thus confirmatory diagnosis relies on postoperative pathology (8, 9).

The two cases lacked specific clinical manifestations but presented to the hospital due to left scrotal or inguinal swelling in a month. Thus, the lesions had rapid growth. In addition, both cases lacked any serum tumor markers, such as CEA, AFP, β-HCG and LDH showing no significant abnormalities. Besides, the scrotal US demonstrated irregular and isoechoic mass, while the Color Doppler US image showed significant vascular signals. Both the CT scan and MRI showed localization of the tumor in the paratesticular region, with no definite distal metastasis. Thus, to improve diagnosis precision, we suggest that adolescents should be educated about self-examination. A thorough physical examination is necessary at the discovery of the painless scrotum or inguinal enlargement. Besides, US and MRI should be the main methods to examine localization of the lesion. They are simple and do not emit any radiation. Further, we recommend preoperative UNB for rapid confirmatory diagnosis. Some previous case reports did not present preoperative diagnosis of the paratesticular RMS prior to the first surgery and underwent simple resection of the lesion. The cases underwent remedial radical operation after post-operative pathologic reports. In recent years, testicular and epididymal puncture has been accepted as a safe and reliable diagnostic method, which is not associated with the risk of tumor needle implantation or tumor metastasis (10). In our case report, preoperative UNB using coaxial needle was adopted to confirm the pathological diagnosis. It not only facilitates comprehensive preoperative MDT discussion and treatment regimen, but enables radical resection or RPLND at the initial operation, avoiding multiple surgeries and reducing metastasis or recurrence. Besides, it protects the male reproductive function and improves the quality of life.

### Histopathological Characteristics

According to the international RMS classification, the common histological subtypes are alveolar, embryonal, botryoid embryonal, spindle cell embryonal, and anaplastic (11). Embryonal RMS (eRMS) is the most frequent type, which accounts for 60% of the cases. On the other hand, paratesticular RMS is rare in adults but often occurs in children, representing 7 to 10% of the tumors in male genitourinary system (12). Thus, since it’s uncommon in adults, few studies have reported paratesticular eRMS (13, 14). Pathologically, the eRMS presents the existence of poorly differentiated cells and rhabdomyoblasts, with abundant eosinophilic cytoplasm indicating embryonal rhabdomyosarcoma. Cytogenetically, the eRMS is characterized by loss of heterozygosity on the short arm of chromosome 11 (15). Whereas, electron microscopy and chromosome analysis are useful methods to improve the pathological diagnosis of the eRMS; they were not examined in our cases.

Histopathologically, the two cases showed a pale profile of gross specimen with fish-like appearance. Microscopically, the tumor comprised of a significant number of rhabdomyoblasts with elongated eosinophilic cytoplasmic tales, commonly referred to as tadpole cells or strap cells. In addition, there were cases of cross-striations within the cytoplasm. The lesion presented a storiform growth pattern with abundant collagen between the tumor cells. It showed an arrangement of tumor cells in bundles with a low to moderate amount of collagen, resembling a leiomyosarcoma. Immunohistochemical findings showed the expression of MyoD1, Myogenin and Desmin in the tumor cells, while keratin and CD34 were negative. On the basis of these findings, a final diagnosis of spindle cell variant of the rhabdomyosarcoma was conducted. The peripheral resection margin and both seminal vesicles did not show presence of tumor cells. Besides, there was no vessel or perineural invasion.

### Individualized Treatment Regimens

The RMS prognosis is related to age, histology, lesion site, and tumor diameter (16, 17). The 5-year survival rate is 22.2% in eRMS patients with recurrence and metastasis and 94.6% in those without metastasis (18). The prognosis in adult patients is worse than that in children, with a 5-year tumor-free survival and 5-year overall survival rates of 28 and 40% respectively, in adults (19).

We, therefore, suggest that there should be a MDT discussion among specialties from the surgery department, endocrinology department, reproductive center and other departments after confirmation of the eRMS. A comprehensive and individualized, but optimal, treatment plan should include tumor resection and preservation of sexual and reproductive functions of the male patients.

Surgical excision should be extensive and thorough. Paratesticular RMS often extends beyond the visual margin, and therefore microscopic examination of the excision margin might yield positive results. Therefore, surgery should be performed with extensive and thorough excision, and a precise circumstantial histopathological examination should be performed to determine the presence of microscopic tumor residues. For the patients with a high risk of local recurrence or positive margin, it is recommended to undergo reoperation of the positive margin area or perform adjuvant radiotherapy (20, 21). Although radical operation was performed in Case 2, local tumor recurrence occurred 1 year after operation. The recurrence was due to the large size of the primary tumor, insufficient scope of resection, lack of postoperative pathological examination of the resection margin, and lack of administration of adjuvant chemoradiotherapy in the abdominal and tumor bed area.

Nevertheless, the role of RPLND has always been controversial (22, 23) and any consensus tends to abandon lymphadenectomy (24, 25). RPLND is not only a therapeutic modality but also an established staging for patients. However, the postoperative complications depend on different surgeons. From our experience, as for eRMS, RPLND should be determined based on tumor staging and preoperative imaging (CT, MRI). RPLND is not preferred for clinical stage I–II patients with no retroperitoneal lymph node metastasis, and is thus substituted with retroperitoneal radiotherapy. In Case 1, the primary tumor was completely resected and further underwent chemoradiotherapy, which could better control the micro-metastasis and local recurrence. On the other hand, the patient in Case 2 experienced local recurrence after 1 year following the operation, but the CT scan showed no lymph node enlargement and no metastasis in the retroperitoneal lymph node dissection. For patients with stage III–IV clinical stage and suspected metastasis of the enlarged retroperitoneal lymph node, unilateral nerve-sparing RPLND is recommended to decrease the risk of relapse and preserve antegrade ejaculation. In Case 2, sexual function can be restored after the RPLND operation.

Moderate chemotherapy and radiation therapy are also recommended. On the basis of imaging and histopathology, patients with stage I clinical stage were treated with the standard VAC regimen (vincristine, actinomycin D and cyclophosphamide), which was effective in the treatment of pediatric rhabdomyosarcoma (1). Retrospective analysis of the survival factors related to RWS found that tumor diameter of >5 cm and the diagnosis age of >10-years old had a poor prognosis, and adjuvant chemotherapy was the main preventive measure to recurrence (26). On the other hand, Fagundes et al. observed that adjuvant radiotherapy could reduce local recurrence (27). After local irradiation of spermatic sarcoma, there was 100% inhibition of recurrence (28). Thus, we believe that recurrence and metastasis of the eRMS are similar across cancer types and the main goal for treatment is to prevention of recurrence and metastasis. Adjuvant radiotherapy and sequential or cyclic chemotherapy should aid extensive radical resection. As for the Case 1, the patient was in clinical stage I because no signs of metastasis were found in the CT scan. After radical resection, pediatric VAC adjuvant chemotherapy regimen with six cycles (6 months) and adjuvant radiotherapy for retroperitoneal area (30 Gy) and tumor bed (20 Gy) were applied. The data demonstrated lack of local recurrence or metastasis, with curative effect. In the second case, the patient had local relapse largely due to non-utilization of adjuvant chemoradiotherapy after first-time surgery. A complete surgery interference of the recurrent tumor was performed and medium dose (30 Gy) radiotherapy in the tumor bed was administered after operation, which achieved a satisfactory curative effect.

### Diagnosis, Treatment, and Follow-Up of the Reproductive Endocrine Function

Paratesticular RMS often occurs in children and young adults and usually has a better prognosis and higher survival rate after radical tumor resection, compared with other RMS (29). Previous reports have shown that the 5-year survival rates were 97 and 84% for patients diagnosed at the age of <10- and >10-year-old respectively (30). In other words, it is an important issue for long-term survivors to restore and sustain their fertility and other endocrine functions. In fact, surgery, chemotherapy and radiotherapy are likely to have some negative impacts on the reproductive endocrine function of patients. Therefore, the treatment scheme should be evaluated in the first-time MDT discussion.

We suggest sperm cryopreservation before chemoradiotherapy to prepare for artificial insemination and assisted reproduction as in Case 2. Secondly, retroperitoneal lymph nodes should be treated with a higher dose (30 Gy) radiotherapy instead of RPLND to avoid possible complications of surgery. In addition, long-term follow-up of hormone function tests and personal mental health is required. For instance, in Case 2, spermatogenic Chinese medicine named Guilingji, HCG treatment scheme and sildenafil were administered 3 months after the surgery. Indeed, the patient gradually restored his erectile function after 9 months following the surgery. Besides, his semen returned to normal after 39 months following the operation, aiding the early recovery of male sexual function and quality of life.

## Conclusion

Due to the rapid growth of adult paratesticular RMS, there is need for timely confirmatory diagnosis and comprehensive treatment plan. Preoperative ultrasound-guided needle biopsy is an available option for rapid diagnosis. Besides, extensive and thorough surgical excision is key to the treatment. Radiotherapy or retroperitoneal lymph node dissection used based on the tumor clinical stage. The adult chemotherapy regimen can refer to pediatric VAC regimen. Sperm cryopreservation should be performed before radiotherapy and chemotherapy. Treatment and follow-up of reproductive endocrine function after surgery can significantly improve the quality of life.

## Ethics Statement

The studies involving human participants were reviewed and approved by Jinhua Hospital Affiliated to Zhejiang University School of Medicine. The patients/participants provided their written informed consent to participate in this study. Written informed consent was obtained from the individuals for the publication of any potentially identifiable images or data included in this article.

## Author Contributions

ZWZ and YX collected all the clinical and pathological data. YZ analyzed and interpreted the patient data and was a major contributor in writing the manuscript. ZSZ revised the final manuscript. All authors contributed to the article and approved the submitted version.

## Funding

This work was supported by grants from Zhejiang Provincial Technological Research Project for Public Welfare (grant number LGF18H050006).

## Conflict of Interest

The authors declare that the research was conducted in the absence of any commercial or financial relationships that could be construed as a potential conflict of interest.
